# *Spathelia belizensis*, a new species and first record for the genus in Central America (tribe Spathelieae, Rutaceae)

**DOI:** 10.3897/phytokeys.75.10015

**Published:** 2016-12-14

**Authors:** Pedro Acevedo-Rodríguez, Steven W. Brewer

**Affiliations:** 1 Department of Botany, National Museum of Natural History, Smithsonian Institution, P.O. Box 37012, Washington, D.C. 20013-7012, U.S.A.; 2 Copperhead Environmental Consulting, Inc., 11641 Richmond Rd. P.O. Box 73 Paint Lick, KY 40461

**Keywords:** Belize, Golden Stream Corridor Preserve, identification key, Spathelieae

## Abstract

*Spathelia* L. as currently circumscribed is endemic to the western portion of the West Indies, and contains nine species, one endemic to the Bahamas, three endemic to Jamaica and five endemic to Cuba. The discovery of a new species in Belize brings the total number of species in *Spathelia* to ten and expands its known distribution beyond the West Indies. *Spathelia belizensis* **sp. nov.** is herein described, illustrated and contrasted to its most morphologically similar congener. A key to the species of *Spathelia* is provided.

## Abstract (es)

El género *Spathelia* es en la actualidad circunscrito como endémico de la porción occidental de las Antillas con un total de nueve especies, una endémica de las Bahamas, tres endémicas de Jamaica y cinco endémicas de Cuba. El descubrimiento de una nueva especie en Belice amplía el número total de especies a diez y expande la distribución del género más allá de las Antillas. En este artículo *Spathelia belizensis* es descrita e ilustrada y se compara con el congénere morfológicamente más similar. También, se proporciona una clave para las especies de *Spathelia*.

## Introduction

The genus *Spathelia* L. (tribe Spathelieae, Rutaceae) is characterized by palm-like trees with unbranched, slender trunks, and a distal crown of spirally arranged, compound leaves. The trees are reported as monocarpic, i.e., producing a distal massive inflorescence after six to eight years and dying right after fruiting. As currently circumscribed, *Spathelia* is considered endemic to the western portion of the West Indies and closely related to *Dictyoloma* and *Sohnreyia*, two monospecific South American genera (Appelhans et al. 2011, Pirani and Groppo 2015).

While carrying out ecological surveys in the Golden Stream Corridor Preserve in Belize, the junior author discovered a population of trees that refers to an undescribed species of *Spathelia*. This discovery documents for the first time the occurrence of *Spathelia* s.s. outside the West Indies, i.e., the northern, eastern tip of Central America. Previous reports of *Spathelia* (i.e., *Spathelia rhoifolia* DC.) from Mexico are referred to the anacardiaceous genus *Pseudosmodingium*.

The study of this novel collection from Belize reveals a new species which seems to be closely related to *Spathelia brittonii* P. Wilson due to morphological similarities. The new species, *Spathelia belizensis* is herein described, illustrated and contrasted to *Spathelia brittonii* its putative closely related species. A key to the species of *Spathelia* is presented to facilitate the identification of the species.

## Material and methods

The description of the new species is based on a single collection, field notes and photographs obtained by the junior author. The study is based on a morphological comparative study of the species of *Spathelia* as recognized by Beurton (2008) and indicates that the Belizean collection is an undescribed species in the genus. A key to the species is based on various floristic treatments of the Antilles (Marie-Victorin 1948, Adams 1972, Correll and Correll 1982, Beurton 2008) and the examination of specimens when necessary. The type specimens are composed of two sheets, one bearing leaf material and the other bearing portions of the fruiting inflorescence. The holotype is deposited in US herbarium, and the isotypes were distributed to K and NY herbaria (acronyms follow Index Herbariorum; http://sweetgum.nybg.org/science/ih/).

## Taxonomic treatment

### Key to the species of *Spathelia*

**Table:** 

1	Sepals almost as long as the petals	**2**
–	Sepals half as long as or shorter than the petals	**8**
2	Leaflets 140–200 per leaf	***Spathelia splendens* (Cuba)**
–	Leaflets 24–92 per leaf	**3**
3	Leaflets long acuminate	***Spathelia glabrescens* (Jamaica)**
–	Leaflets obtuse, acute or truncate	**4**
4	Plant glabrous or puberulous	**5**
–	Plant stellate pubescent, especially along young parts	**6**
5	Flowers white	***Spathelia bahamensis* (Bahamas)**
–	Flowers red	***Spathelia wrightii* (Cuba)**
6	Trees 6–16 m tall; leaflets 7–12 cm long; flowers pinkish lilac to magenta	***Spathelia simplex* (Jamaica)**
–	Treelets 1–3 m tall; leaflets 1.5–7 cm long; flowers red	**7**
7	Leaflets 25–57 per leaf; sepals ovate; fruits emarginate at apex	***Spathelia cubensis* (Cuba)**
–	Leaflets 60–92 per leaf; sepals linear; fruits obtuse or sub-emarginate at apex	***Spathelia vernicosa* (Cuba)**
8	Treelets ca. 3 m tall, glabrous; leaflets entire or crenulate	***Spathelia coccinea* (Jamaica)**
–	Trees to 16 m tall, indument of stellate and simple hairs; leaflets crenate or crenate-dentate	**9**
9	Leaflets crenate-dentate, 42–65 per leaf; sepals of equal size; petals 5, bright red to pink; fruits obovate and marginate at apex	***Spathelia brittonii* (Cuba)**
–	Leaflets crenate, 75–91 per leaf; sepals of unequal sizes; petal (?–) 6, white; fruits ovate and obtuse at apex	***Spathelia belizensis* (Belize)**

### Spathelia belizensis

Taxon classificationPlantaeSapindalesRutaceae

Acev.-Rodr. & S.W. Brewer sp. nov.

urn:lsid:ipni.org:names:77159070-1

Figs 1 , 2

#### Diagnosis.

The new species *Spathelia belizensis* seems to be closely related to *Spathelia brittonii* P. Wilson due to their similar adult height, large leaves, and indument of stellate and simple hairs. *Spathelia belizensis* differs from the latter by having leaves with 75–91 leaflets with crenate margins, unequal sepals, white petals, and fruits with ovate outline and obtuse apex.

#### Type.

Belize; Toledo District. Northern portion of the Golden Stream Corridor Preserve. Upper slope of a limestone ridge with rocky, clay-loan soil, 151 m, 16°24'13"N, 88°47'28"W, 26 Feb 2014 (fl,fr), *S.W. Brewer & G. Stott 7110* (holotype: US [US01863784 & US01863785]; isotypes: K, NY).

#### Description.

Tree up to 16 m tall; trunk single, unbranched, ca. 15 cm DBH, which tapers towards the ferruginous stellate distal portion; leaf scars numerous; bark greyish brown to tan–grey with ridges flat and shallow, regularly anastomosing forming a *diamond pattern* on the lower 2/3 of the trunk, slash light-colored. Leaves imparipinnate with elliptic-oblanceolate outline, 100–110 × 19–22 cm, alternate to spirally arranged on distal portion of stem forming a dense crown; petiole and rachis slightly flattened along adaxial surface, ferruginous stellate pubescent, intermixed with whitish, strigose trichomes, petioles 10–15 cm long; leaflets ca. 75–91, opposite for the most part, alternate towards the distal portion of the leaf, oblong-lanceolate, 4–12 × 1.5–2.5 (gradually smaller toward both ends of the leaf), sessile, with slightly asymmetrical, rounded/cordate base, long acuminate at apex, margins revolute, crenate (sinuate in basal leaflets), with numerous oil glands, adaxial surface sparsely stellate except for the densely stellate midvein, abaxial surface sparsely strigose especially along the prominent midvein, and sparsely stellate. Inflorescence a distal frondo-bracteate, terminal, panicle-shaped thyrse, > 1 × 1 m. Flowers in loose botryoid cymes; pedicels strigose, 5–9 mm long; calyx green, slightly fleshy, strigose, sepals unequal, oblong-lanceolate, 1.2–1.5 mm long, with an enlarged apical gland; petals 6, white, oblong-ovate, obtuse at apex with an apical gland; filaments (pistillate flowers) ca. 1.5 mm long, wingless or shortly winged at base, setulose on lower half; gynoecium oblong, trigonous, ca. 4 mm long, strigose; stigma subglobose, yellowish, nearly sessile. Fruit strigose, trigonous-winged, with ovate outline and obtuse apex, turning from green to reddish brown; endocarp slightly woody, with large cavity on dorsal side.

**Figure 1.**
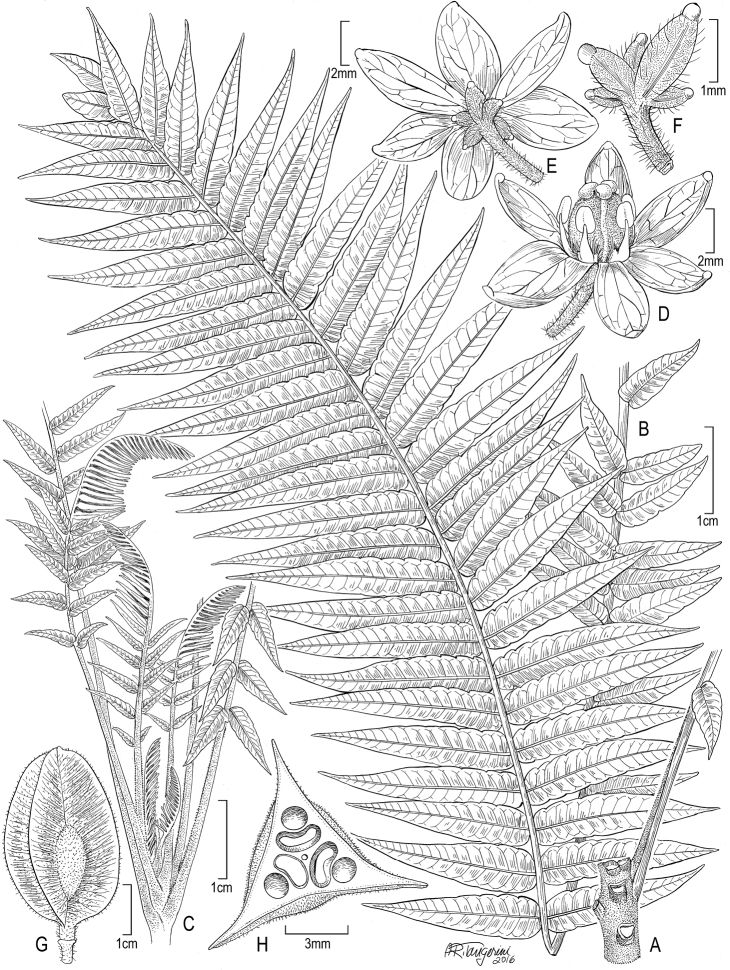
*Spathelia belizensis*. **A** Portion of distal branch **B** Leaf **C** Distal portion of branch showing flush of new leaves **D** Flower, frontal view **E** Flower basal view **F** Calyx **G** Fruit **H** Cross section of fruit. By Alice Tangerini based on *Brewer & Stott 7110* (US).

**Figure 2.**
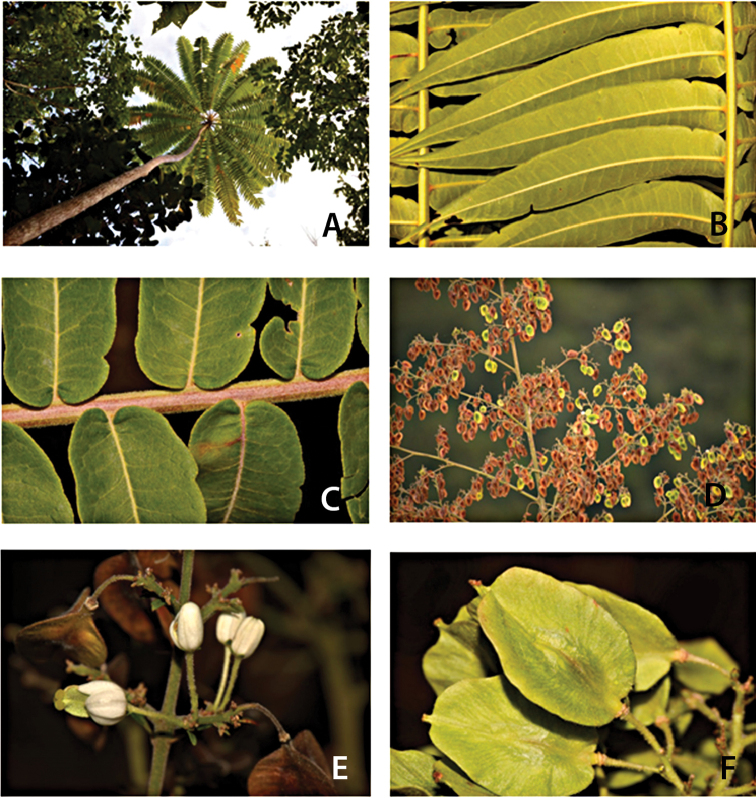
*Spathelia belizensis*. **A** Habit showing crown of leaves **B** Middle portion of leaf showing abaxial surface of leaflets **C** Portion of leaf rachis showing basal portion of leaflets **D** Portion of infructescence **E** Botryoid cyme showing flowers and fruits **F** Young fruits. Pictures by S. Brewer, based on *Brewer & Stott 7110* (US).

#### Distribution and ecology.

Known only from the northern portion of the Golden Stream Corridor Preserve and adjacent portions of the Columbia River Forest Reserve in Belize; on steep slopes of Cretaceous limestone hills at elevations of c. 80–250 m.

#### Specimens examined.

Only the type collection was studied.

#### Etymology.

The specific epithet refers to the country where the new species is known to occur.

### Conservation status

*Spathelia belizensis* appears to be limited to the Golden Stream Corridor Preserve in Belize where several individuals have been spotted by the junior author. However, in the absence of precise information about its frequency the species can only be treated as DD (Data deficient) within IUCN guidelines.

## Discussion

*Spathelia belizensis* and *Spathelia brittonii* seem closely related as they share similar heights, large leaves, and indument of stellate and simple hairs. Nevertheless, *Spathelia belizensis* differs from the latter by having leaves with 75–91 leaflets (vs. 42–65), crenate leaflets (vs. crenate-dentate), unequal sepals (vs. equal), petals 6 and white (vs. 5 and bright red to pink), and fruits with ovate outline and obtuse apex (vs. obovate and marginate at apex).

The type specimens of *Spathelia belizensis* contained only 6-merous flowers, a feature that departs from other species of *Spathelia* as they are known to have only 5-merous flowers. Because this collection only had few flowers, it is premature to regard the presence of 6-merous flowers as a distinctive character of the new species. Future collections may show it also to have 5-merous flowers.

## Supplementary Material

XML Treatment for Spathelia belizensis
